# Tea Polyphenols Inhibit the Occurrence of Enzymatic Browning in Fresh-Cut Potatoes by Regulating Phenylpropanoid and ROS Metabolism

**DOI:** 10.3390/plants13010125

**Published:** 2024-01-02

**Authors:** Yuge Guan, Sainan Lu, Yan Sun, Xinrui Zheng, Run Wang, Xinghua Lu, Linjiang Pang, Jiyu Cheng, Lei Wang

**Affiliations:** 1 School of Food and Health, Zhejiang Agricultural and Forestry University, Hangzhou 311300, China; 2 MOE Key Laboratory of Macromolecular Synthesis and Functionalization, Department of Polymer Science and Engineering, Zhejiang University, Hangzhou 310058, China; 3 Zhejiang-Israel Joint Laboratory of Self-Assembling Functional Materials, ZJU-Hangzhou Global Scientific and Technological Innovation Center, Zhejiang University, Hangzhou 311215, China

**Keywords:** tea polyphenols, fresh cut, phenylpropanoid, reactive oxygen species, Vc-GSH cycle, quality

## Abstract

During fresh-cut processing, potatoes lose their inherent protective cellular structure, leading to enzymatic browning that compromises sensory and edible quality. Tea polyphenols (TPs), natural preservatives with potent reducing properties, are hypothesized to impact this browning process. However, their influence and regulatory mechanism on the enzymatic browning of fresh-cut potatoes remain poorly understood. This study used the “Holland Seven” potato as the research material to explore the effects of a treatment with different TP concentrations (0.1 g L^−1^, 0.2 g L^−1^, and 0.3 g L^−1^) on the browning phenomenon and quality of fresh-cut potatoes during storage. The results showed that appropriate concentrations of TP treatment had a good preservation effect on the appearance and edible quality of fresh-cut potatoes. Furthermore, exogenous TP treatment reduced the content of enzymatic browning substrates (caffeic acid, p-coumaric acid, and ferulic acid) by regulating phenylpropanoid metabolism. Meanwhile, TP treatment augmented the activities of antioxidative enzymes (superoxide dismutase, catalase, ascorbate peroxidase, and glutathione reductase), maintained higher levels of ascorbic acid (Vc), and reduced glutathione (GSH). Consequently, the TP treatment could inhibit enzymatic browning by regulating reactive oxygen species (ROS) metabolism and the Vc-GSH cycle in fresh-cut potatoes.

## 1. Introduction

Potato (*Solanum tuberosum* L.), a major global staple crop, is extensively cultivated worldwide and serves as an important cash crop [1]. Recently, the growing popularity of fast and convenience foods has steadily increased potato consumption, spurring the rapid development of fresh-cut potatoes [2]. Fresh-cut potatoes, also known as minimally processed potatoes or semi-processed potatoes, refer to ready-to-eat or ready-to-use products. These potatoes undergo a series of processes such as grading, cleaning, trimming, peeling, cutting, preservation, and packaging, and then they are transported at low temperatures and sold in refrigerated cabinets. They are characterized by being natural, nutritious, healthy, clean, fresh, and convenient, and are increasingly favored by consumers. Fresh-cut potatoes expand the application scope of potato raw materials, facilitate their comprehensive utilization, and represent a breakthrough in the potato industry. Since 2015, China has been promoting a strategy to establish potatoes as a staple food. Fresh-cut potatoes are poised to play a vital role in sustainable agriculture, food security, and enhancing the nutritional diversity of diets [3]. However, during fresh-cut processing, potatoes lose their protective cellular tissues, resulting in damage, accelerated browning, water loss, and epidermal hardening, significantly affecting their sensory and product quality [4]. Browning, a major issue in their processing and storage, is a key factor limiting their shelf life. This browning occurs when cut potatoes come into contact with phenoloxidase and substrates in aerobic conditions, leading to the formation of melanin [5]. In addition, cutting leads to an outbreak of reactive oxygen species (ROS) within the tissues, exacerbates membrane lipid peroxidation, and causes a decrease in potato’s cells, such as a decrease in antioxidant enzyme activity and nonenzyme antioxidants content, which may also further accelerate enzymatic browning [6].

Phenolic compounds, as the main substrates for enzymatic browning, play an important role in the enzymatic browning process of fresh-cut fruits and vegetables. In plants, phenolic compounds are mainly synthesized through the phenylpropanoid metabolic pathway. Therefore, regulating the phenylpropanoid metabolism is an effective method to delay enzymatic browning in fresh-cut fruits and vegetables. Research by Dai et al. showed that sodium nitroprusside can control enzymatic browning of fresh-cut potatoes by inhibiting the activity of the key enzyme phenylalanine ammonia-lyase (PAL) in the phenylpropanoid metabolic pathway, thereby reducing the synthesis rate of phenolic compounds [7]. Treatment with exogenous proline and sodium bisulfite reduces phenolic compounds in fresh-cut potatoes and enhances antioxidant enzyme activity, thereby preserving them against enzymatic browning [8,9]. However, the application of chemical browning inhibitors is limited due to potential food safety concerns [10]. Therefore, finding safe preservation technology that can effectively inhibit the browning of fresh-cut potatoes has important theoretical significance and application value.

Recent studies have shown that plant extracts have shown great potential in preventing browning [11]. For example, onion and garlic extracts inhibit polyphenol oxidase (PPO) activity due to their high content of sulfur compounds, thereby inhibiting browning [12,13]. Water extract of purslane (0.05%, *w*/*w*) effectively inhibits PPO, peroxidase (POD), and PAL activities and also regulates nonenzymatic ROS scavenging systems (ascorbic acid, glutathione, polyphenols, etc.) to enhance the antioxidant capacity, thereby inhibiting the browning of fresh-cut potatoes during storage [14]. Tea polyphenols (TPs), nontoxic and safe antioxidants, are extensively used as nutritional fortifiers in the functional food industry [15]. Recently, with food industry advancements, TPs have emerged as a novel natural food preservative [16,17].

Previous research found that TPs could improve sensory quality and microbiological safety, delayed weight loss, and extended the shelf life of fresh-cut products [18,19,20,21]. However, the application of TPs on fresh-cut potatoes have not yet been reported, and how TPs regulate enzymatic browning needs to be studied. Additionally, the optimal concentration for treatment is still unclear, limiting their industry application. Based on the above scientific questions, this study aims to investigate the effect of exogenous TP treatment on enzymatic browning in stored fresh-cut potatoes. It dynamically monitors browning values, determines the optimal tea polyphenol concentration for browning inhibition, and explores the interactions between key enzyme activities, metabolic products, antioxidant enzymes, and antioxidant substances. The study seeks to elucidate the physiological and biochemical roles of phenylpropanoid and ROS metabolism in enzymatic browning and lay a theoretical foundation for developing new preservation techniques for fresh-cut fruits and vegetables.

## 2. Results and Discussion

### 2.1. Browning Index and Appearance

**Figure 1:**
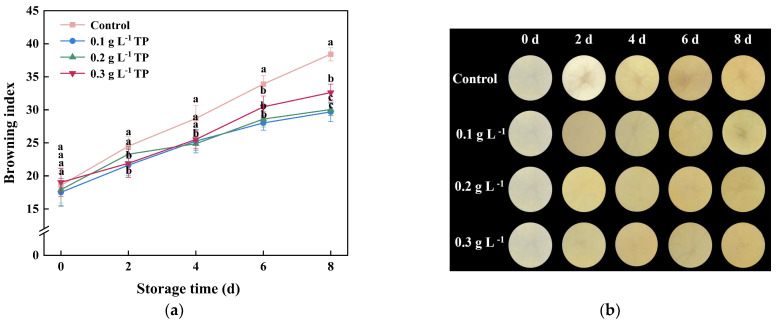
Browning index (**a**) and appearance (**b**) of fresh-cut potatoes treated with TPs during 8 d of storage at 4 °C. Values with different letters were significantly different at *p* < 0.05.

Fresh-cut potatoes are widely favored by consumers. However, enzymatic reactions triggered by tissue damage during cutting lead to rapid oxidative browning in the presence of oxygen [22]. As shown in Figure 1a, the browning index of fresh-cut potatoes gradually increased with storage time. After 2 days of storage, the browning value of the control group increased by 31.93% compared to the initial day. Throughout storage, TP treatment consistently yielded a lower browning index than the control. This effect is likely due to TPs’ robust antioxidative properties, enabling them to scavenge free radicals and inhibit browning [23]. However, higher TP concentrations could provide additional substrates for the oxidative browning reaction, thus intensifying the browning in products [24]. Notably, in the later stages of storage, lower concentrations of TPs (0.1 and 0.2 g L^−1^) more effectively prevented browning than the higher concentration (0.3 g L^−1^).

To directly observe the quality of fresh-cut potatoes, photographs were taken throughout the storage period (Figure 1b). From day 2, the control group displayed a progressive browning, whereas the tea polyphenol-treated group maintained a superior appearance quality. These results align with previous studies on fresh-cut burdock [25], dragon fruit [20], and lotus root [21] where TPs, as natural antioxidants, effectively curbed enzymatic browning in fresh-cut produce by neutralizing excessive ROS produced during processing, thereby decelerating oxidation rates. The result of Figure 1b indicated that the appearance quality corresponded to the browning index results.

### 2.2. Weight Loss Rate and Firmness

**Figure 2:**
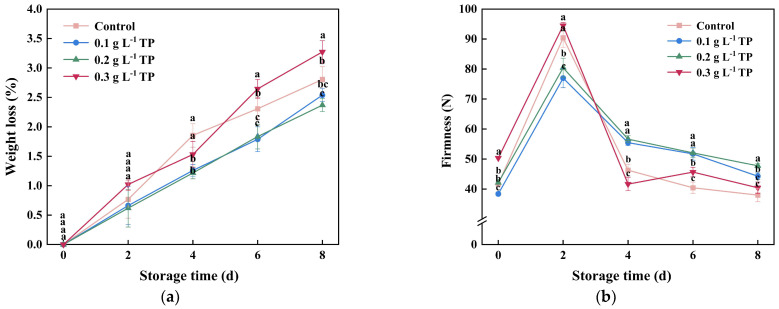
Weight loss (**a**) and firmness (**b**) of fresh-cut potatoes treated with TP during 8 d of storage at 4 °C. Values with different letters were significantly different at *p* < 0.05.

Weight loss is a crucial metric for assessing fresh-cut potato quality. Figure 2a revealed a gradual increase in weight loss with extended storage for all groups. On the eighth day, the weight loss was 2.80% for the control group and 2.54%, 2.37%, and 3.27% for the 0.1, 0.2, and 0.3 g L^−1^ TP-treated groups, respectively. The highest concentration (0.3 g L^−1^) significantly increased weight loss, especially in later stages, where it was 14.67% and 6.79% higher than the control. This increase could stem from accelerated metabolic processes and water loss induced by the 0.3 g L^−1^ tea polyphenol treatment [26]. It is worth noting that 0.1 and 0.2 g L^−1^ TP-treated potatoes exhibited consistently lower weight loss rates than the control, suggesting that appropriate concentration of TP treatment effectively delayed the weight loss of fresh-cut potatoes.

As shown in Figure 2b, the firmness of fresh-cut potatoes rapidly increased in the early stage of storage. On the 2nd day, the firmness of the control group and the 0.1, 0.2, and 0.3 g L^−1^ TP-treated group increased to 2.17, 2.01, 1.91, and 1.87 times that of original values, respectively. This increase is likely due to the formation of wound-healing tissues at the mechanically damaged sites, enhancing surface firmness [27]. It is worth noting that similar to the weight loss results, the hardness values of the 0.3 g L^−1^ TP-treated group were higher than those of the control group in the first 2 days of storage. This could be due to the high concentration of TPs causing an imbalance in osmotic pressure between the internal and external environments of the cell membrane, leading to a significant substance leakage from the cells and accelerated tissue aging, resulting in higher weight loss and firmness [28]. After two days, firmness in all groups declined, with the 0.1 and 0.2 g L^−1^ TP groups showing significantly lower values than the control and 0.3 g L^−1^ TP groups. This result suggests that lower TP concentrations effectively preserve the firmness of fresh-cut potatoes during storage.

The above results showed that the impact of the TP treatment on browning was concentration-dependent, with the 0.2 g L^−1^ and 0.1 g L-1 concentrations exhibiting more pronounced inhibitory effects. Notably, the 0.2 g L^−1^ and 0.3 g L^−1^ TP treatments effectively maintained antioxidant levels (Vc and GSH) in fresh-cut potatoes. However, the higher concentration (0.3 g L^−1^) negatively influenced weight loss and hardness. Overall, the 0.2 g L^−1^ TP treatment emerged as the optimal choice for preserving the edible quality of fresh-cut potatoes. However, TPs are a mixture composed of various monomeric phenols, and they may have certain impacts on the flavor and taste of food. For instance, phenolic substances like catechins and polymeric tannins may induce astringency and bitterness in taste. Volatile phenolic compounds could generate specific aromas. Therefore, the future application of tea polyphenols in the field of fresh-cut potato preservation requires further exploration of their potential influence on product flavor.

### 2.3. Phenolic Content

As the important antioxidants in fresh fruits and vegetables, phenolic compounds play a significant role in antioxidation, and they also have therapeutic and preventive effects against chronic diseases such as obesity, heart disease, and cancer [29,30]. Moreover, they are substrates of the browning reaction in fresh-cut fruits and vegetables. To further elucidate the mechanism of tea polyphenols in inhibiting browning in fresh-cut potatoes, this study analyzed the content of phenolic compounds in potatoes.

**Figure 3:**
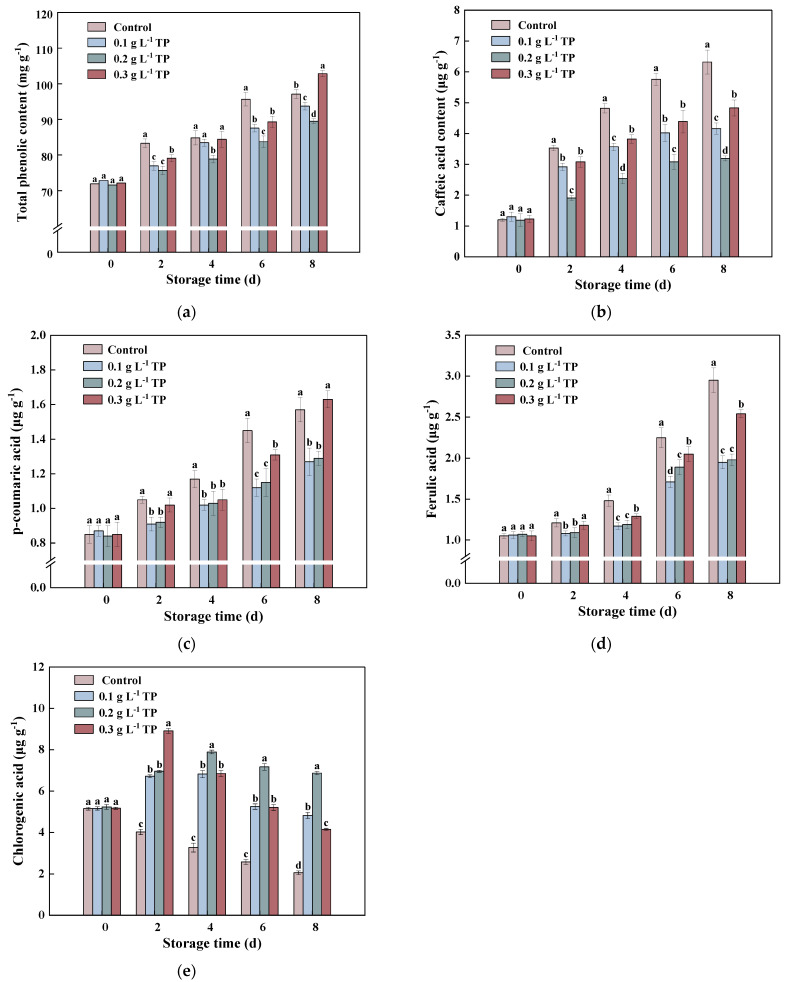
Total phenolic content (**a**), caffeic acid (**b**), p-coumaric acid (**c**), ferulic acid (**d**), and chlorogenic acid (**e**) content of fresh-cut potatoes treated with tea polyphenols during 8 d of storage at 4 °C. Values with different letters were significantly different at *p* < 0.05.

As shown in Figure 3a, fresh-cut treatment significantly increases phenolic compound accumulation in potatoes, a finding echoed in studies on fresh-cut broccoli [31], fresh-cut celery [32], fresh-cut onion [33], and fresh-cut carrot [34]. This indicates that fresh fruits and vegetables initiate secondary metabolic processes and synthesize phenolic compounds to counteract mechanical damage, thereby rapidly restoring metabolic balance [35]. In the early storage period, the control group had the highest total phenolic content, 33.01% higher on the sixth day compared to before storage. By the eighth day, the 0.3 g L^−1^ TP-treated group’s total phenolic content peaked, significantly higher than other groups, indicating that high-concentration TP treatments can suppress the increase in total phenolic content in fresh-cut potatoes over a short period, while lower concentrations have a continuous inhibitory effect. Among the different treatments, the 0.2 g L^−1^ TP treatment group had the best effect in inhibiting the increase in phenolic substance content in fresh-cut potatoes.

Due to the variety of phenolic substances in potatoes and the distinct oxidation characteristics of each, this study quantitatively analyzed the four main monomeric phenols in fresh-cut potatoes using an HPLC-DAD. According to Figure 3b, the content of caffeic acid rapidly increased, with the control group showing significantly higher levels than the treatment groups (*p* < 0.05). Caffeic acid levels in the 0.3 g L^−1^ TP group were significantly greater than in the 0.1 g L^−1^ and 0.2 g L^−1^ TP groups (*p* < 0.05).

The p-coumaric acid content in fresh-cut potatoes increased rapidly throughout the storage period (Figure 3c). p-coumaric acid increased more slowly in the 0.1 g L^−1^ and 0.2 g L^−1^ TP groups, remaining lower than in the control group. No significant difference in p-ferulic acid content was observed between the 0.1 g L^−1^ and 0.2 g L^−1^ TP groups. On the 8th day, p-coumaric acid levels in the 0.3 g L^−1^ TP group were significantly higher than in the control group (*p* < 0.05).

As shown in Figure 3d, the content of ferulic acid in all four experimental groups showed an increasing trend. Throughout the storage period, the content of ferulic acid in the control group was significantly higher than that in the TP treatment (0.3 g L^−1^). The high-concentration TP treatment (0.3 g L^−1^) had a higher ferulic acid content compared to the low-concentration TP treatment (0.1 g L^−1^ and 0.2 g L^−1^).

In contrast to the trends observed for the other three monomeric phenols, the content of chlorogenic acid in fresh-cut potatoes of the control group decreased throughout the entire storage period (Figure 3e). The content of chlorogenic acid in the tea polyphenol treatment groups showed an initial increase followed by a decrease. The content of chlorogenic acid in the 0.3 g L^−1^ TP treatment group decreased after 2 days of storage, while the content of chlorogenic acid in the 0.1 g L^−1^ and 0.2 g L^−1^ TP treatments decreased after 4 days of storage. The chlorogenic acid content in the 0.2 g L^−1^ TP treatment group was the highest compared with the other groups.

**Figure 4:**
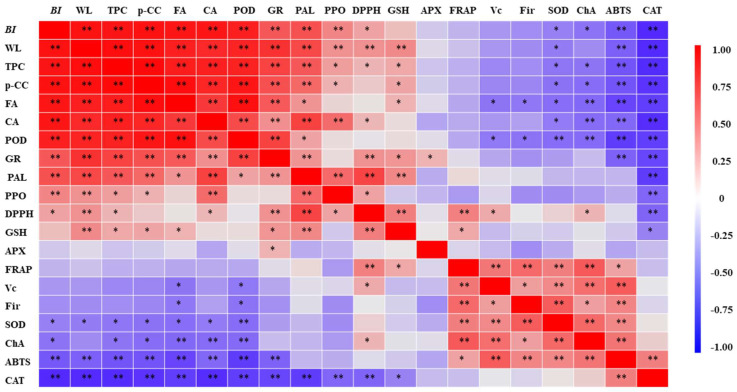
Pearson correlation matrix for each index. * *p* < 0.05, ** *p* < 0.01. BI, browning index; WL, weight loss; TPC, total phenol content; p-CC, p-coumaric acid; FA, ferulic acid; CA, caffeic acid; POD, peroxidase; GR, glutathione reductase; PAL, phenylalanine ammonia-lyase; PPO, polyphenol oxidase; GSH, glutathione; APX, ascorbate peroxidase; FRAP, ferric-reducing antioxidant power; Fir, firmness; SOD, superoxide dismutase; ChA, chlorogenic acid; CAT, catalase.

A correlation analysis (Figure 4) revealed a significant positive correlation between caffeic acid, p-coumaric acid, ferulic acid, and the browning index, with correlation coefficients above 0.90. Conversely, chlorogenic acid showed a significant negative correlation with browning. These findings indicate that these phenolic compounds play pivotal roles in enzymatic browning, with caffeic acid, p-coumaric acid, and ferulic acid promoting browning and chlorogenic acid inhibiting it. The variation in oxidative characteristics and enzymatic specificity in different fruits and vegetables influences this process [36]. For instance, in broccoli, sinapic acid and caffeic acid are optimal substrates for enzymatic browning [37], whereas in apples, chlorogenic acid is more prone to oxidation [38]. In longan [39] and lychee [40], epicatechin directly contributes to enzymatic browning. The study demonstrates that TP treatment maintains chlorogenic acid content, curbs the rapid accumulation of other phenolics, and thus delays browning in fresh-cut potatoes.

Because phenolic substances are an important factor affecting the taste and flavor of food [38], the future challenges of the TP application in commercial fresh-cut potatoes primarily involve two aspects. On one hand, a further in-depth exploration is needed to understand the impact of TP treatment on the sensory attributes, flavor, and quality of fresh-cut products. On the other hand, a thorough investigation into the underlying mechanisms of how TP treatment influences the quality of fresh-cut potatoes is essential. In the future, phosphoproteomics and transcriptomics techniques could be used in exploring the regulation mechanism of TP on phenylpropane metabolism and enzyme-catalyzed browning pathways. This, in turn, will offer new insights for optimizing preservation techniques.

### 2.4. Key Enzyme Activities in Phenolic Synthesis and Oxidative Decomposition

**Figure 5:**
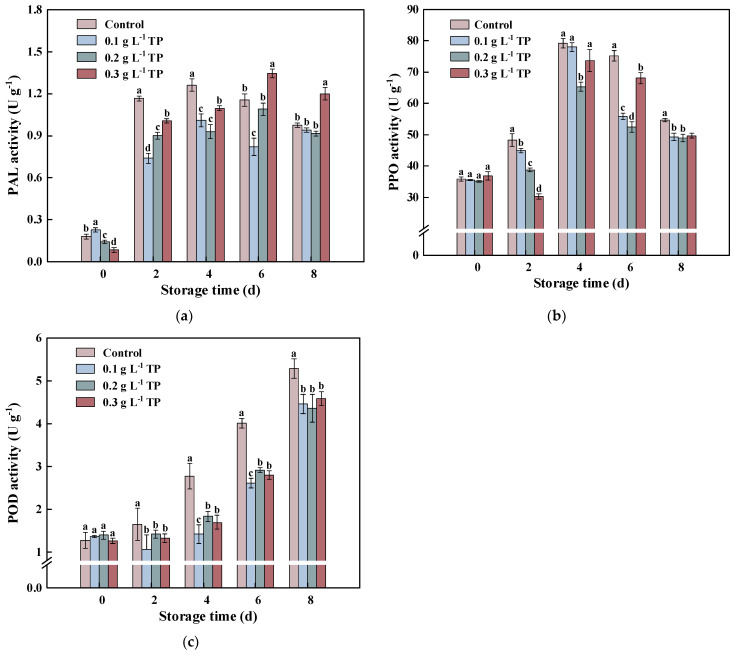
Phenylalanine ammonia-lyase (PAL) (**a**), polyphenol oxidase (PPO) (**b**), and peroxidase (POD) (**c**) activity of fresh-cut potatoes treated with tea polyphenols during 8 d of storage at 4 °C. Values with different letters were significantly different at *p* < 0.05.

PAL is a crucial rate-limiting enzyme in the phenylpropane metabolic pathway, playing a vital role in stress- and mechanical-damage-induced defense responses. PAL participates directly in the biosynthesis of phenolic compounds at damaged sites, aiding in protection and healing [35]. As shown in Figure 5a, the PAL activities in the four experimental groups initially increased and then decreased. The PAL activities in the 0.1 and 0.2 g L^−1^ TP groups were consistently lower than the control, while the 0.3 g L^−1^ TP group exhibited a higher activity after 6 days. These results suggest that a low-concentration TP treatment consistently inhibits PAL activity in fresh-cut potatoes, whereas a high-concentration treatment is effective only in the early stages. In the long term, low-concentration TPs are more effective. A correlation analysis showed significant positive correlations between PAL activity and both total phenol content and browning index (coefficients of 0.764 and 0.736, respectively), suggesting that the TP treatment regulates PAL activity, affecting phenolic synthesis and inhibiting browning in fresh-cut potatoes.

PPO and POD are critical phenolic substrate-dependent oxidoreductases in plants, catalyzing enzymatic browning. Figure 5b shows that fresh-cut treatment induced a rapid increase in PPO activity, peaking on the fourth day of storage. The TP treatment effectively reduced PPO activity throughout storage, with the 0.3 g L^−1^ TP group showing the best initial inhibitory effect and the 0.2 g L^−1^ TP treatment being the most effective after four days. The PPO activity in all groups significantly decreased later in storage, likely due to increased tissue aging. Figure 5c demonstrates that the POD activity in all treatment groups continually increased throughout storage, with the TP treatment consistently lowering POD activity.

Normally, intact fruits and vegetables resist enzymatic browning as PPO, POD, and phenolic compounds are in separate cellular compartments, preventing a direct interaction between oxidases and substrates [41]. However, mechanical damage in fresh-cut produce disrupts this separation, increasing the contact between polyphenols and atmospheric oxygen, leading to a phenolic oxidation catalyzed by PPO and POD. This process results in a dark brown substance formation and accumulation, causing enzymatic browning. This study found consistently lower PPO and POD activities in TP-treated potatoes than in the control group, with both activities positively correlated with the browning index. This suggests that the TP treatment delayed enzymatic browning in fresh-cut potatoes by inhibiting these enzymes. Contrarily, in fresh-cut broccoli, a methyl jasmonate (MeJA) treatment increased POD activity while inhibiting browning, highlighting POD’s dual biological functions [37]. Besides its role in oxidative browning, POD catalyzes the hydrogen peroxide decomposition, maintaining plant tissue membrane integrity and resisting oxidative damage from reactive oxygen species [42,43]. Thus, TPs regulate both synthesis and oxidation processes of phenolic compounds and then delay browning in fresh-cut potatoes during storage.

### 2.5. Antioxidant Enzyme Activity

Enzymatic browning in fresh-cut fruits and vegetables is an oxidation reaction influenced by plant antioxidant enzymes that regulate the ROS metabolism [44]. Among them, superoxide dismutase (SOD), a crucial antioxidant metalloenzyme, catalyzes the dismutation of O_2_^−^ to generate O_2_ and H_2_O_2_ [45]. As shown in Table 1, the overall SOD activity in the four experimental groups showed an initial increase followed by a decrease. On the 2nd day of storage, the SOD activity in the 0.1 g L^−1^, 0.2 g L^−1^, and 0.3 g L^−1^ TP-treated groups was 2.08-fold, 3.03-fold, and 1.83-fold higher than the untreated group, respectively. Throughout the storage period, the 0.1 g L^−1^ and 0.2 g L^−1^ TP-treated groups maintained a higher SOD activity than the control, indicating the TP treatment could enhance the SOD activity in fresh-cut potatoes. Catalase (CAT), another important oxidoreductase, decomposes H_2_O_2_ into O_2_ and H_2_O, scavenging free radicals and protecting organisms from oxidative damage [46]. Contrary to the SOD activity, the CAT activity in fresh-cut potatoes decreased throughout storage, TP-treated groups exhibited a higher CAT activity than the control, and the CAT activity in 0.2 g L^−1^ TP-treated groups was the highest.

The ascorbate-glutathione (Vc-GSH) cycle, crucial for redox reactions in plants, involves key enzymes like ascorbate peroxidase (APX) and glutathione reductase (GR), which significantly clear ROS [47]. As shown in Table 1, the APX activity in the control group, 0.1 g L^−1^, and 0.3 g L^−1^ TP-treated groups exhibited a repeated process of decline and increase. However, the 0.2 g L^−1^ TP-treated group’s APX activity decreased initially, then rapidly increased from days 2 to 6, stabilizing between days 6 and 8. On the 8th day, the APX activity ranked as follows: 0.2 g L^−1^ > 0.3 g L^−1^ > 0.1 g L^−1^ > control. Unlike the APX activity, the GR activity in all groups increased over the storage period, with TP-treated groups showing a higher activity than the control (Table 1). By day 8, there was a significant difference in GR activity among the treatment groups (*p* < 0.05), with the GR activity in the control, 0.1 g L^−1^, 0.2 g L^−1^, and 0.3 g L^−1^ groups increasing by 2.61-fold, 3.71-fold, 4.41-fold, and 3.46-fold compared to day 0, respectively. The above results revealed that 0.2 g L^−1^ TP treatment could effectively maintain SOD, CAT, APX and GR enzyme activities.

Different fruits and vegetables exhibit varying responses in their antioxidant enzyme systems to mechanical damage after fresh-cut processing. For instance, the CAT activity in fresh-cut broccoli [44] and pumpkin [48] showed an increase in early storage, opposite to this study’s findings. The upward trends in SOD and GR activities in fresh-cut dragon fruit [49] align with this study. However, unlike in dragon fruit, the APX activity in fresh-cut potatoes showed dynamic and irregular changes during storage. These disparities arise because oxidative damage from fresh-cut processing involves complex signal transduction processes regulated by multiple injury pathways. These pathways interact, transmitting and activating defense gene transcription and expression, leading to self-defense and repair against oxidative damage in plants [50]. Therefore, specific responses of antioxidant enzyme systems may vary based on plant species, tissue type, and storage conditions.

### 2.6. Antioxidants Content

Ascorbic acid, also known as vitamin C (Vc), is primarily found in fresh fruits and vegetables and is an important antioxidant in plant-based foods. Glutathione (GSH) is a tripeptide that contains a thiol group and possesses strong antioxidant capabilities. Vc and GSH are antioxidants involved in the Vc-GSH cycle. Vc can generate dehydroascorbate through the oxidation action of APX and reduce H_2_O_2_, effectively eliminating free radicals in cells. GSH can generate Vc through the catalytic action of dehydroascorbate reductase (DHAR), thus complementing each other in the process of scavenging reactive oxygen species [51]. Therefore, this experiment further measured the content of antioxidants (Vc and GSH) in fresh-cut potatoes.

**Figure 6:**
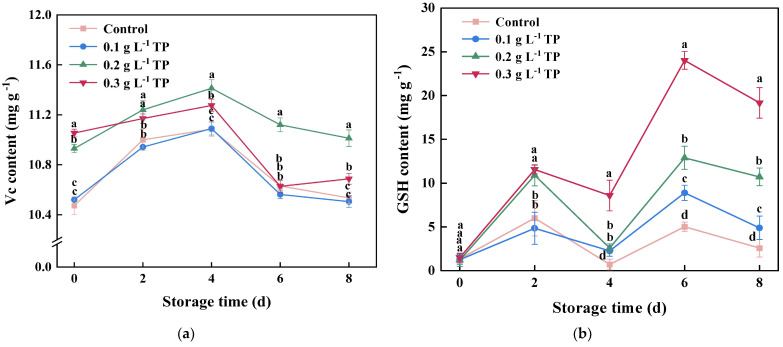
Vc (**a**) and GSH (**b**) contents of fresh-cut potatoes treated with tea polyphenols during 8 d of storage at 4 °C. Values with different letters were significantly different at *p* < 0.05.

According to Figure 6a, the Vc content initially rose and then fell, peaking after four days of storage. The Vc content in the 0.2 g L^−1^ TP-treated group consistently exceeded that of the control group, indicating that different concentrations of TP treatment had different effects on the Vc content in fresh-cut potatoes. Conversely, the GSH content exhibited fluctuations, peaking on the second and sixth days (Figure 6b). After the sixth day, the GSH levels in TP-treated groups significantly surpassed the control group. At the end of the storage period (eight days), the GSH content in the 0.3, 0.2, and 0.1 g L^−1^ TP-treated groups was 7.45, 4.17, and 1.90 times higher than that in the control group, respectively. Similar studies have been reported in fresh-cut dragon fruit [52] and fresh-cut broccoli [44]. Additionally, different fruits and vegetables with diverse biological characteristics employ different defense mechanisms against oxidative damage induced by mechanical injury. For example, fresh-cut apples [52] accumulate Vc near the wound site to prevent melanin formation, while the ascorbic acid content in strawberries [53] and oranges [54] gradually decreases after fresh-cut processing. The ascorbic acid content in dragon fruit [49] shows no significant changes. These findings suggested that TP treatment can regulate antioxidant synthesis and breakdown in the Vc-GSH cycle by modulating the Vc and GSH contents.

### 2.7. Antioxidant Capacity

**Figure 7:**
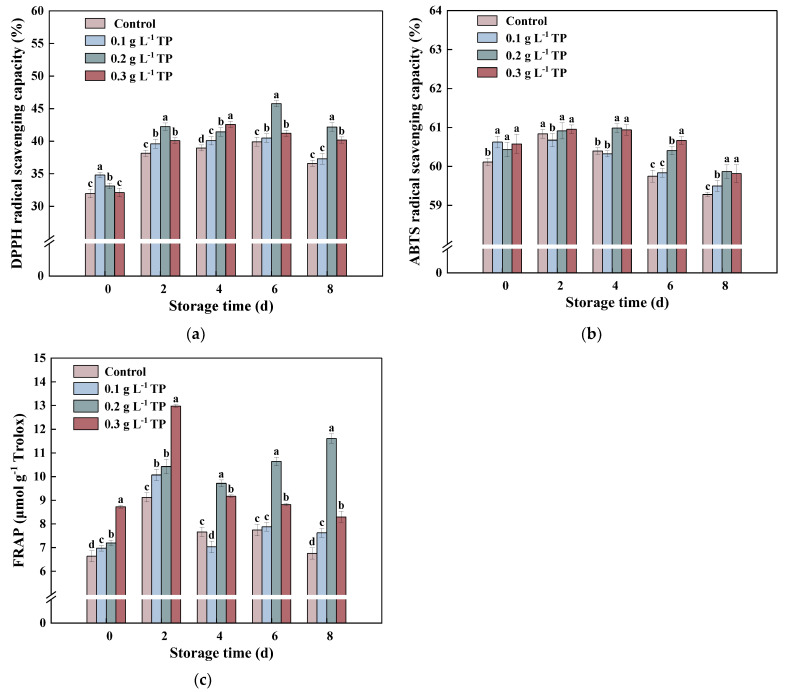
DPPH radical scavenging capacity (**a**), ABTS^+^ radical scavenging capacity (**b**), and ferric-reducing antioxidant power (FRAP) (**c**) of fresh-cut potatoes treated with tea polyphenols during 8 d of storage at 4 °C. Values with different letters were significantly different at *p* < 0.05.

Figure 7a–c present bar graphs for 2,2-diphenyl-1-picrylhydrazyl (DPPH), 2,2-Azinobis-3-ethylbenzthiazoline6-sulphonate (ABTS), and ferric ion reducing antioxidant power (FRAP) assays, respectively, illustrating the antioxidant capacity of fresh-cut potatoes under various conditions. DPPH is a stable radical, and the presence of hydrogen-donating antioxidants can neutralize its unpaired electron, eliminating free radicals. Figure 7a reveals that all experimental groups initially showed an increased DPPH radical scavenging capacity which later decreased but remained higher at each storage interval compared to day 0. By the eighth day, the scavenging capacity order was 0.2 g L^−1^ TP > 0.3 g L^−1^ TP > 0.1 g L^−1^ TP > control, indicating that the TP treatment significantly enhanced fresh-cut potatoes’ DPPH radical scavenging ability. This result is consistent with previous research that the addition of polyphenols in food could make it significantly resistant to oxidation [30,55].

Like DPPH, ABTS is a stable radical, and its scavenging capacity also first increased, then decreased, as shown in Figure 7b. There was no significant difference in ABTS scavenging capacity between the 0.1 g L^−1^ TP group and the control group during storage. The 0.2 g L^−1^ TP and 0.3 g L^−1^ TP-treated groups showed a higher ABTS scavenging capacity than the 0.1 g L^−1^ TP group. This suggests that different concentrations of TP treatment significantly impact the ABTS scavenging capacity in fresh-cut potatoes.

As shown in Figure 7c, the peak value of FRAP appeared on the 2nd day of storage. The FRAP peaks of the 0.3 g L^−1^, 0.2 g L^−1^, 0.1 g L^−1^ TP-treated groups, and the control group were 1.49, 1.45, 1.44, and 1.38 times higher than at 0 days, respectively. Consistent with the results of the DPPH radical scavenging capacity, the 0.2 g L^−1^ TP-treated group exhibited the highest FRAP value at the end of storage.

A correlation analysis (Figure 4) revealed that of the four kinds of phenolic compounds in fresh-cut potatoes, only chlorogenic acid significantly correlated positively with the antioxidant capacity (ABTS, DPPH, FRAP). The other compounds (caffeic acid, ferulic acid, and p-coumaric acid) showed varying degrees of positive and negative correlations with the antioxidant capacity. This is mainly because the structure of phenolic substances could affect antioxidant properties [30]. This aligns with Wolfe et al., who found that chlorogenic acid had a greater cellular antioxidant activity than other phenolics like caffeic acid in HepG-2 cell-based assays [56]. Additionally, Vc and GSH were significantly positively correlated with antioxidant capacity (DPPH, FRAP), suggesting they also enhance fresh-cut potatoes’ antioxidant capacity. This contrasts with findings in fresh-cut broccoli, where no significant correlation existed between antioxidant capacity and Vc or GSH. This discrepancy could be due to differing biological traits; broccoli synthesizes phenolics in response to mechanical injury, countering oxidative damage, whereas potatoes use various antioxidants to manage mechanical stress [57]. Additionally, ABTS exhibited a significant negative correlation with the browning index, suggesting that TPs enhanced the ability to scavenge free radicals, inhibit browning, and regulate the phenylpropane metabolism, Vc-GSH cycle, and antioxidant enzyme system in fresh-cut potatoes. It is worth noting that the TP treatment effectively delayed the enzymatic browning phenomenon, whereas it enhanced the antioxidant capacity. The results show that a treatment with TPs may be a convenient way to obtain more health-promoting, physiological, and antioxidant activities from fresh-cut potatoes, whether commercially or at home.

## 3. Materials and Methods

### 3.1. Plant Material and Pretreatment

Potatoes, specifically the Holland No. 7 variety, were purchased from Hema Fresh, a local market in Hangzhou. Fresh, mature, similarly sized potatoes, free of diseases, pests, and mechanical damage, were chosen for the experiment. Tea polyphenols (TPs, food grade, purity 98%) were obtained from Shanghai Yuanye Biochemical Co., Ltd. (Shanghai, China). Initially, potatoes were washed with tap water to remove soil residues. After drying, they were peeled and manually sliced into 1 cm thickness. Based on the preliminary experimental results, the samples were then soaked in TP solutions of 0.1 g L^−1^, 0.2 g L^−1^, and 0.3 g L^−1^ concentrations for 2 min. Samples immersed in distilled water for 2 min served as the control group. Treated samples, after air-drying in a sterile ventilated cabinet, were placed in plastic trays (100 g/box), sealed with polyethylene film, and stored at 4 °C for 8 days. Physicochemical parameters were measured bidaily.

### 3.2. Colorimetric Values and Appearance

The color of fresh-cut potatoes was represented by L*, a*, and b* values, where L* indicates brightness, a* indicates red-green color, and b* indicates yellow-blue color. The L*, a*, and b* values of each sample were measured using a CR400 colorimeter. Based on the measured L*, a*, and b* values, the browning index (BI) of fresh-cut potatoes was calculated using the following Equations (1) and (2) [58], and photographs presented the changes in visual quality of fresh-cut potatoes during storage.

(1) $$\;\;x=\frac{\left(a^{*}+1.75\right)}{\left(5.646L^{*}+a^{*}-3.012b^{*}\right)}$$

(2) $$\;\;BI=\frac{\left(x\;-0.31\right)}{0.172}\times 100$$

### 3.3. Weight Loss and Hardness Determination

The weight loss of fresh-cut potatoes was calculated using the mass difference method [51]. The weight loss formula is Weight Loss = (*W*_0_ − *W_t_*)/*W*_0_ × 100%, where *W*_0_ is the initial weight (g), and *W_t_* is the weight at each storage time (g).

The hardness of fresh-cut potatoes was measured using a TA.XT Plus texture analyzer (Stable Micro Systems Co., Ltd., London, UK) with a 5 mm diameter cylindrical probe [4]. The probe penetrated the potato at 1 mm s^−1^ to a depth of 6 mm, measuring hardness in Newtons (N).

### 3.4. Determination of Total Phenolic and Monomeric Phenolic Content

A 5.0 g sample was mixed with 20 mL of 80% ethanol and underwent ultrasonic extraction at 40 °C in darkness for 40 min. Following centrifugation at 12,000× *g* for 30 min, the total phenolic extract was acquired. Subsequently, 1 mL of extract was combined with 1 mL of Folin–Ciocalteu reagent, and 10 mL of 7% Na_2_CO_3_ was added within 8 min. The mixture was then brought to a volume of 25 mL with deionized water. After incubating for 90 min at room temperature in darkness, the supernatant’s absorbance at 750 nm was measured [59]. The total phenolic (TP) content was expressed as mg g^−1^, which was calculated with the formula *TP* = *n* × *V/m*, where *n* is the phenolic content (mg mL^−1^) from the gallic acid standard curve, *V* is the extraction solution’s total volume (mL), and *m* is the sample weight (g).

High-performance liquid chromatography (HPLC) was used to analyze four monomeric phenolic compounds, including caffeic acid, ferulic acid, p-coumaric acid, and chlorogenic acid, in potatoes [3]. The chromatographic conditions were as follows: Agilent 1260 UV detector, C_18_ reverse-phase column (250 × 4.6 mm, 5 μm), column temperature of 25 °C, injection volume of 20 μL, flow rate of 0.8 mL min^−1^, gradient elution with methanol (A) and a 1% formic acid water solution (B), with the following elution conditions: 0–10 min, 75–60% A; 10.01–60 min, 60–40% A; 60.01–65 min, 40–75% A. The detection wavelength was set at 280 nm, and the content of each monomeric phenolic compound was calculated based on the respective standard curves (μg g^−1^).

### 3.5. Determination of PAL Enzyme Activity

The determination of PAL enzyme activity referred to the method by Liu et al. [60]. Five grams of potato samples was added to 20 mL of extraction solution (0.1 mol L^−1^ pH 8.8 borate-boric acid buffer). The mixture was centrifuged at 4 °C and 12,000× *g* for 30 min, and the supernatant was collected. In a test tube, 3 mL of 50 mM pH 8.8 borate buffer and 0.5 mL of 20 mmol L^−1^ L-phenylalanine were mixed. Following a 10 min preincubation at 37 °C, 0.5 mL of enzyme solution was added. The initial absorbance at 290 nm (OD_0_) of the mixture was then quickly measured. After a 60 min incubation at 37 °C, the final absorbance at 290 nm (OD_1_) was measured. The PAL activity was quantified by the change in absorbance before and after incubation, with one unit defined as a 0.01 increase in absorbance at 290 nm per hour.

### 3.6. Determination of PPO and POD Enzyme Activity

Five grams of samples was immersed in 20 mL of pH 6.4 phosphate buffer. The homogenized mixture was then centrifuged at 12,000× *g* for 30 min, and the supernatant was collected to measure PPO and POD activities. The PPO activity assay involved mixing 0.5 mL of enzyme extract with 3 mL of 0.5 M catechol solution. The absorbance of the reaction was measured at 398 nm within 30 s to calculate the PPO activity [61].

The determination of the POD activity referred to Singh et al. [62]. In brief, 0.5 mL enzyme extract was added to 2 mL of 0.05 mol L^−1^ pyrogallol solution. After incubating at 30 °C in a water bath for 5 min, 1 mL of 0.08% H_2_O_2_ solution was added, and the change in absorbance at 460 nm was measured within 60 s. The enzyme activity was expressed as U g^−1^.

### 3.7. Determination of Antioxidant Substance Content

For the ASA extraction: mix 0.15 g of fresh-cut potatoes with 0.45 mL of extraction agent, vortex, then centrifuge at 8000× *g* for 10 min the supernatant to determine the ASA content. For the GSH extraction: combine 0.1 g of sample with 1 mL of extraction solution and centrifuge at 8000× *g* for 10 min; the supernatant serves as the GSH extract. The ASA and GSH content determination followed the reagent kit (Article Number: ASA-1-W; Article Number: GSH-1-W) instructions from Suzhou Cominbio Biotechnology Co., Ltd. (Suzhou, China); the results are expressed as mg g^−1^.

### 3.8. Determination of Antioxidant Enzyme Activity

The extraction method for CAT was identical to that of POD. The CAT assay involved 0.5 mL of enzyme extract and 3 mL of 20 mM H_2_O_2_. The absorbance at 240 nm was scanned for 180 s [63]. A change in absorbance of 0.01 per gram per minute defined one unit (U) of CAT activity. The CAT activity was expressed in units per gram (U/g).

The SOD activity determination followed the reagent kit instructions (Article Number: SOD-1-Y): Mix 0.1 g of potatoes with 1 mL of extraction solution, homogenize in an ice bath, and centrifuge at 8000× *g*, 4 °C for 10 min. The supernatant serves as the test solution. Prepare the reaction mixture as per the instructions and measure the absorbance at 560 nm. One unit of SOD activity is defined when the inhibition in the xanthine oxidase reaction system reaches 50%.

The APX activity was determined with slight modifications according to Li et al.’s method [64]: Mix 5.0 g of potatoes with 20 mL of pH 7.5 phosphate buffer, homogenize in an ice bath, and centrifuge at 12,000× *g*, 4 °C for 30 min. Combine 0.1 mL of the supernatant with 2.6 mL of reaction solution and 0.3 mL of 2 mM H_2_O_2_ solution. Measure the absorbance at 290 nm within 180 s after mixing thoroughly. The calculation of the APX activity was identical to that of CAT, expressed in units per gram (U g^−1^).

The GR activity determination followed the reagent kit instructions (Article Number: GR-1-W) from Suzhou Cominbio Biotechnology Co., Ltd.: Take 0.2 g of potato sample, add 1 mL of extraction solution, and centrifuge at 8000× *g*, 4 °C for 15 min; the collected supernatant is used for testing. One unit of GR activity is defined as the catalysis of 1 nmol NADPH oxidation per gram per minute.

### 3.9. Determination of Antioxidant Capacity

This study used ABTS and DPPH radical scavenging assays, along with ferric-reducing antioxidant power (FRAP), to assess the antioxidant capacity of fresh-cut potatoes. The extraction method for the ABTS and DPPH assays followed the previously mentioned total phenolic extraction process [65]. The reaction procedures for the ABTS and DPPH assays were based on methods from our previous research. Briefly, the ABTS assay involved mixing 20 μL of extract with 80 μL of ABTS solution. Following a 6 min dark incubation at 20 °C, the absorbance at 734 nm was measured. The DPPH assay combined 100 μL of extract with 100 μL of DPPH solution. After a 30 min incubation at 20 °C, the absorbance at 517 nm was measured with a microplate reader. The ABTS and DPPH assay results were expressed in percentages. The FRAP assay followed instructions (FRAP-1-G) from Suzhou Cominbio Biotechnology Co., Ltd. Results were expressed in μmol g^−1^ Trolox.

### 3.10. Data Analysis

All experiments were conducted in triplicate, with each data point representing the average of three trials. Microsoft Excel 2016 was utilized for organizing data and calculating averages and standard deviations. Data visualization was performed using Origin 7.5. A one-way ANOVA tested the significance of differences, with multiple comparisons using the least significant difference (LSD) method. The correlation test was conducted with IBM SPSS 20 (IBM Corp., Armonk, NY, USA), and Pearson’s correlations among each index were analyzed. The significance level was set at *p* < 0.05.

## 4. Conclusions

In conclusion, a 0.2 g L^−1^ TP treatment emerged as the optimal choice for preserving the appearance, color, hardness, and other qualities of fresh-cut potatoes. Moreover, the 0.2 g L^−1^ TP treatment played a crucial role in inhibiting PAL activity, leading to a delay in phenolic compound synthesis. Consequently, the levels of specific phenolic acids decreased, resulting in a 7.91% reduction in total phenolics. Additionally, the 0.2 g L^−1^ TP treatment effectively inhibited PPO activity, delayed phenolic oxidation, and enhanced the activities of antioxidant enzymes (SOD, CAT, APX, and GR). This comprehensive approach led to a substantial improvement in DPPH and FRAP capacities, with a remarkable increase of 15.26% and 71.85%, respectively, compared to the control. In summary, the 0.2 g L^−1^ TP treatment emerges as a promising strategy for inhibiting browning, preserving edible quality, and enhancing antioxidant capacity in fresh-cut potatoes. Thus, these findings not only provide a new strategy for the storage of fresh-cut potatoes but also offer feasible industrial application pathways for the preservation of other fresh-cut products that are prone to browning.

## Figures and Tables

**Table 1:** Superoxide dismutase (SOD), catalase (CAT), ascorbate peroxidase (APX) and glutathione reductase (GR) activities of fresh-cut potatoes treated with tea polyphenols during 8 d of storage at 4 °C. Values with different letters were significantly different at *p* < 0.05.

Storage (d)	Treatment	SOD Activity (U g^−1^)	CAT Activity (U g^−1^)	APX Activity (U g^−1^)	GR Activity (U g^−1^)
0	Control	9.05 ± 0.01 a	10.72 ± 0.31 c	13.20 ± 0.62 c	13.40 ± 0.54 b
	0.1 g L^−1^	8.19 ± 0.00 d	10.96 ± 0.26 c	14.48 ± 0.53 b	13.40 ± 0.54 b
	0.2 g L^−1^	8.76 ± 0.02 c	12.08 ± 0.63 b	16.27 ± 0.50 a	13.94 ± 1.07 b
	0.3 g L^−1^	9.47 ± 0.02 a	13.10 ± 0.08 a	14.58 ± 0.78 b	16.08 ± 1.52 a
2	Control	9.24 ± 0.02 d	6.96 ± 0.02 b	11.76 ± 0.44 c	14.65 ± 2.02 d
	0.1 g L^−1^	19.26 ± 0.02 b	7.99 ± 0.33 a	13.29 ± 0.36 a	19.65 ± 1.82 c
	0.2 g L^−1^	28.01 ± 0.08 a	7.83 ± 0.29 a	12.34 ± 0.18 b	24.30 ± 2.20 a
	0.3 g L^−1^	16.87 ± 0.03 c	8.00 ± 0.40 a	12.79 ± 0.46 ab	23.23 ± 0.51 b
4	Control	5.72 ± 0.01 d	5.08 ± 0.22 c	13.83 ± 0.45 b	24.12 ± 1.61 b
	0.1 g L^−1^	11.35 ± 0.05 b	6.16 ± 0.34 b	13.25 ± 0.28 b	33.77 ± 1.61 a
	0.2 g L^−1^	15.73 ± 0.01 a	7.56 ± 0.10 a	16.47 ± 0.38 a	29.66 ± 4.49 ab
	0.3 g L^−1^	9.16 ± 0.05 c	7.62 ± 0.18 a	11.42 ± 0.35 c	27.34 ± 3.75 b
6	Control	7.39 ± 0.02 c	4.13 ± 0.05 c	12.98 ± 0.38 c	30.55 ± 0.54 c
	0.1 g L^−1^	10.60 ± 0.02 b	4.74 ± 0.32 b	17.66 ± 0.39 a	40.74 ± 0.88 a
	0.2 g L^−1^	10.99 ± 0.20 a	6.36 ± 0.27 a	17.64 ± 0.41 a	37.16 ± 3.31 ab
	0.3 g L^−1^	6.73 ± 0.01 d	5.17 ± 0.28 b	15.48 ± 0.38 b	34.66 ± 1.01 b
8	Control	4.60 ± 0.02 d	2.36 ± 0.17 c	12.68 ± 0.10 d	35.02 ± 1.01 d
	0.1 g L^−1^	6.25 ± 0.01 c	3.61 ± 0.20 b	13.08 ± 0.35 c	49.67 ± 1.34 c
	0.2 g L^−1^	10.00 ± 0.03 a	4.59 ± 0.33 a	17.59 ± 0.33 a	61.46 ± 1.01 a
	0.3 g L^−1^	6.75 ± 0.17 b	3.96 ± 0.27 b	15.32 ± 0.49 b	55.74 ± 1.75 b

## Data Availability

The datasets used and/or analyzed during the current study are available from the corresponding author on reasonable request.
